# An exploratory study of the application of mindsight in email communication

**DOI:** 10.1016/j.heliyon.2020.e04305

**Published:** 2020-07-15

**Authors:** Suzannah Ogwu, Petia Sice, Shelagh Keogh, Colin Goodlet

**Affiliations:** Department of Computer and Information Sciences, University of Northumbria, Newcastle Ellison Building, Ellison Terrace, Newcastle upon Tyne, NE1 8ST, UK

**Keywords:** Psychology, Information science, Virtual communication, Compassion, Empathy, Mindsight, Email communications

## Abstract

**Research problem:**

Email communication is a type of virtual communication with specific characteristics: - it is a form of written communication; it is asynchronous, i.e. not occurring at the same time for the sender and recipients. Email does not include face to face communication and thus the capacity to develop a sense of connection, shared knowledge and trust are distorted due to the lack of interpersonal cues and may become a problem. Communication is a process where one mind affects another, and it is through the process of communication that individuals develop shared perceptions and coordinate their behaviours. This implies creating social worlds rather than disseminating information between people. The fact that communication is not a mere means of disseminating information but also a means of developing social entities for co-creation of understanding requires that individuals approach communication with a sense of awareness of themselves and others.

**Proposed solutions:**

In this article the theory of Mindsight is proposed as an approach of mindful observation of the act of communication resulting in deeper awareness, reflection and potential impact on behaviour. Although Mindsight has been extensively used in addressing self-awareness and communication in real spaces, there is a gap in the existing relevant literature about the application of Mindsight in virtual/email communication.

**Methods:**

A Mindsight Utility for Virtual Communication (MUVC) was developed for use in engaging with email. The MUVC is a set of exercises enabling users to identify and manage their email habits over a period of time. The utility was developed to engage users in experiencing self-awareness and awareness of others and provides an aid to formulate personal guidelines in email communication. It was implemented with nineteen participants as part of action research allowing each individual to develop their own guidelines grounded in the experience of using the observation practice.

**Data:**

Qualitative data was collected in the form of diaries for a period of 6 days from two group of participants: -university students; - employees of a social enterprise. Ethics approval was granted from Northumbria University prior to data collection. Thematic analysis was used.

**Results:**

The study uncovered potential problems and solution strategies, inhibitors and facilitators of communication. Problems with email were perceived as: not knowing one another, difficulties in connecting, lack of trust, lack of interpersonal clues, reduction in communication quality, emotional and psychological discomfort. Solution strategies included: open-mindedness, empathy, compassion, attention focus, clear language, awareness practice, addressing physical and emotional discomfort.

## 1 Introduction

Virtual communication is any communication that is computer-mediated (Myers, 2007). It is different from face to face communication because the communication interactions happen in a virtual space, where by ‘virtual’ we mean a functionality that is aided by computer networks and where ‘space’ is an interactive setting (Yu and Shaw, 2008, Chandler and Munday, 2011).

Virtual communication is essential to organisations, because the achievement of any business depends on effective communication (Malmelin, 2007). The term virtuality was coined by Jaron Lanier in 1980 to describe how digital facilities translated individuals into new forms of existence in the digital world (Baeva, 2016). That is, activities that were only possible in physical spaces are now obtainable in virtual spaces (Yu and Shaw, 2008; Ale Ebrahim et al., 2009; Sköld, 2011; Kellerman, 2014). Also, diverse activities that were unattainable in physical spaces are now feasible in virtual spaces (Kellerman, 2014). For instance, virtual communication has permitted the synchronic presence of people in both physical and virtual spaces (Yu and Shaw, 2008). This has made life flexible, and people are no longer constrained by time and place to perform certain activities; they can function virtually (Shaw and Yu, 2009). It has paved the way for individuals who live in distant places to be cognitively close to one another (Rodriguez and Ryave, 2002) and has positively altered the way people interact with themselves (Yu and Shaw, 2008). A shift has taken place transposing the traditional or natural communication of face-to-face communication to virtual communication thus altering human existence from real beings to virtual ones and impacting interpersonal relationships (Baeva, 2016).

Email communication is a type of virtual communication with specific characteristics: - it is a form of written communication; it is asynchronous, i.e. not occurring at the same time for the sender and recipients; it generates the so called ‘thread’ automatically generated by the computer program (Prieto-Arranz, et al., 2013). Email does not include face to face communication and thus the capacity to develop a sense of connection, shared knowledge and trust are distorted due to the lack of interpersonal cues and may become a problem (Morgan et al., 2014).

The constant dependence of organisations on electronic communication has led to a rapid increase in the volume of emails received by employees nowadays, which is an issue of concern. It is now reported that information overload is affecting workers (Soucek and Moser, 2010); this increases levels of stress among workers as they receive a significant number of emails (Jerejian et al., 2013; Pignata et al., 2015). Information overload occurs when individuals receive information that is beyond their processing ability (Soucek and Moser, 2010). The problem occurs when there is a distribution of many messages that an individual can cope with; this disrupts workflow (Soucek and Moser, 2010). Similarly, having many random messages reduces people's effectiveness in communication as they have to separate relevant from less essential messages (Gupta et al., 2016).

Researchers have put forward recommendations on how to manage email communication and solutions to overcome stress and overload issues. It was suggested that, people should be taught how to apply techniques that would enable them to manage their emails (Burgess et al., 2005; McMurtry, 2014; Soucek and Moser, 2010). Organisations were advised to provide intervention in the form of messaging filters that helps to separate essential emails from unrequested emails (Jerejian et al., 2013; Freed et al., 2008). Others proposed that companies should produce drafted guidelines on how companies’ emails should be utilised (Soucek and Moser, 2010). Similarly, Pignata et al. (2015) and Jackson et al. (2001) recommended training on email management strategies. Others like, Jerejian et al. (2013) and Liu and Moh (2016) suggested that people embrace the email management techniques of filtering manually and they continuously monitor emails. Thus, there are different types of filtering strategies which include, spam detection (Iyengar et al., 2017) and spam classification (Kumaresan and Palanisamy, 2017). There is a suggestion to do emails at set times to avoid constantly checking emails (Levy, 2016; Kushlev and Dunn, 2015).

The proposed solutions for dealing with email communication focused mainly on the environment and on people's behaviour, but did not address: -the individual's awareness of themselves and others; - the importance of self-awareness and reflection on change of behaviour.

Communication is a process where one mind affects another, and it is through the process of communication that individuals develop shared perceptions and coordinate their behaviours (Hedman and Valo, 2015). This implies creating social worlds rather than disseminating information between people. The fact that communication is not a mere means of disseminating information but also a means of developing social entities for co-creation of understanding requires that individuals approach communication with a sense of awareness of themselves and others.

In this article the theory of Mindsight (Siegel 1999 to 2016) is proposed as an approach of mindful observation of the act of communication resulting in deeper awareness, reflection and potential impact on behaviour. Although Mindsight has been extensively used in addressing self-awareness and communication in real spaces (Siegel, 2010b), there was gap in existing literature about the application of Mindsight in email communication.

A practice of email observation (Mindsight Utility for Virtual Communication via email) was developed by the researchers to engage users in experiencing self-awareness and awareness of others, and reflecting on their own ways and habits of email communication. It was implemented with nineteen participants as part of action research allowing each individual to develop their own guidelines grounded in the experience of using the observation practice.

## 2 Theory of mindsight

The concept of Mindsight was first introduced in psychotherapy by Daniel Siegel, MD (2010). He discovered that when he talked with his patients, they said that they felt ‘felt’ by him (Siegel, 2010, p. 27). They had a sense that he understood what they meant and felt. There was no term to describe this process of feeling ‘felt’ in the field of mental health at the time, and he coined the term Mindsight. Mindsight is the ability to discern sensations, thoughts, and emotions while identifying them as activities of the mind. It is also the ability to become aware of emotions and thoughts of others so we can understand their point of view, which enables our responses to be both compassionate and competent. To this effect, Mindsight enables people to monitor and manage their emotions and thoughts and not to be driven by them (Siegel, 2010). Mindsight can be considered to be a powerful lens through which we can understand our inner lives with more clarity, integrate the brain, and enhance our relationships with others. It is a kind of focused attention that allows us to see the internal workings of our minds.

The theory of Mindsight is focused on understanding the workings of the human mind. The theory defines the mind as an embodied and relational process, emerging from the mutual interconnectedness of the physical, mental, and relational (both human and environmental) domains of human reality. The mind, as an emergent property of the body and relationships, is created within the internal neurophysiological processes and relational experiences. In other words, the mind is a process that emerges from the distributed nervous system extending throughout the entire body, and also from the communication patterns that occur within relationships (Siegel, 2009). Relationships refer to how energy and information are managed and shared among persons (Siegel, 2010); these relationships can be at a personal or at a broader level; for example, communities, countries, etc. (Clinton and Sibcy, 2012).

To put it simply, human connections shape neural connections. Relationships and neural linkages together shape the mind and ‘the brain’ (the embodied nervous system) (Siegel 2009, 2016). The mind and relationships are aspects of one reality and need to be considered together, where the body provides the biological structure for hosting human experience, and the mind is an embodied and relational process that regulates the information and energy flow in the embodied brain and in the relationships with others and the environment (Siegel, 2016). The term ‘embodied brain’ refers to the whole nervous system, not just the brain in the skull. The triangle of well-being shows the nature of the reality of energy and information flow. The mind is a regulatory mechanism of the brain and relationships.

Therefore, the regulation of energy and information flow is achieved through the management of intention and attention (Siegel, 2010b); intention determines the direction of attention. Attention acts like ‘a scalpel,’ as the direction and scope of attention can trigger changes in the brain (neural plasticity) and the communication space of relationships and then further influence our mental activity, brain and relationship patterns in mutual interaction (Sice et al., 2013). The intention of ‘seeing reality’ more clearly and continuously enhancing our awareness and reflection capability in making sense of reality, requires the integration and stabilising of attention in monitoring body sensations, mental activity, and relationships.

In western culture, a heightened state of awareness is often referred to as mindfulness. This terminology is widely accepted in the West, where the state of mindfulness is defined as the opposite of mindlessness, that is, where we are engaging/functioning on autopilot, or merely downloading our mental models, assumptions, and prejudices rather than witnessing present experience as it unfolds (Kabat-Zinn, 2009). The author, therefore, provides an operational working definition of mindfulness as the awareness that emerges through paying attention on purpose, in the present moment, and non-judgmentally to the unfolding of experience moment by moment. It is important to clarify that our understanding of mindfulness as paying attention to experience as it unfolds. This is not only connected to present moment sensations but connected to accepting and witnessing our present moment experience which may involve some or all aspects of experience, that is, sensations, mental activity (thoughts, feelings, memory, intentions, beliefs, attitudes, etcetera) and relational experience (connectedness to others, to our planet, to nature, etc.) (Sice et al., 2013). Therefore, individuals taking part in email communication need some sort of reminder that the connections that exist are beyond mere email communication but are a process of interconnection with others.

## 3 Application of mindsight: the wheel of awarness

The theory of Mindsight is a systems theory grounded in insights from complexity science, systems biology and psychotherapy (Sice et al., 2018). It suggests that health (harmonious functioning) comes from integration: it is the linkages of parts that are different in a system. Different here means that, the components that make up a subset of anything could be different, such as individuals in a family can become distinctive. When these different parts are linked together and linkages are established, integration is promoted. This happens when members of a group can interact with each other, or when the various activities the mind can perform such as feeling and thinking are in the awareness of being connected (Siegel, 2012). Promoting differentiation alone or too much linkage does not promote integration, but the combination of differentiation and linkages can lead to integration (harmonious functioning). A well-integrated mind is characterized by coherence, flexibility, and adaptability and when a mind is not properly integrated, it is characterized by chaos and/or rigidity. That is why it is essential to observe the way an individual can integrate their mind, brain and relationships because integration enhances attuned communication, insight, empathy, body regulation, emotional balance, flexibility, intuition, fear extinction, morality (Siegel, 2007). Attuned communication leads to interpersonal relationships with compassion and empathy.

**Figure 1:**
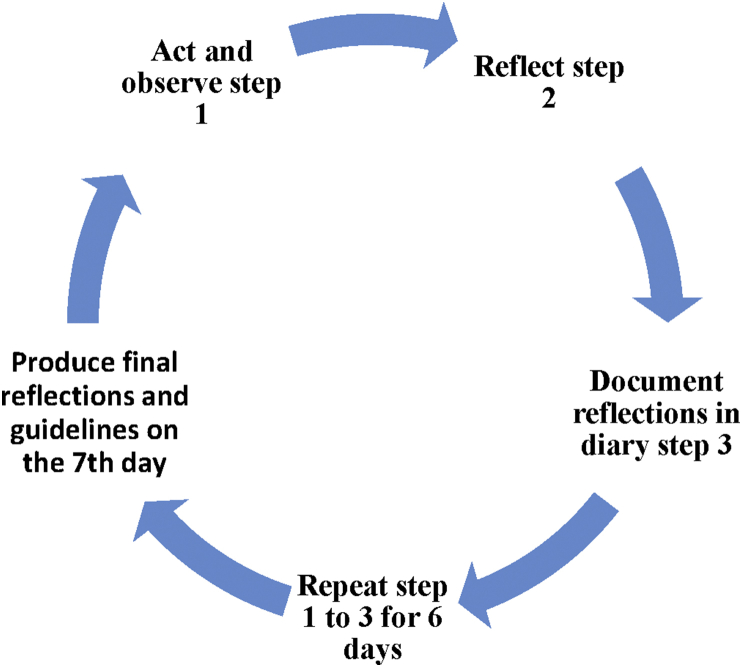
Action research for participants.

Siegel (2007) develops a practice called the Wheel of Awareness (WOA) which seeks to promote integration of the mind. WOA is a symbol of the mind (Figure 1), the hub of the WOA represents an open awareness that can be directed through attention. The wheel spokes connect the hub part to the rim with the exterior part symbolising anything the individual can be aware of. The imaginary wheel enables the person to categorise awareness (Baldini et al., 2014). The hub represents the awareness itself and the spokes are what a person can be aware of: the five senses, the sixth sense, i.e. bodily awareness; the seventh sense – awareness of mental activity and the eight sense – awareness of connectedness with others (Siegel, 2012).

The five senses, sight, hearing, touch smell and taste relate to the environment, the fifth sense is what is known as sensory awareness is the kind of awareness that emerges from the five senses of sight, hearing, smelling, tasting and touching. The interior of the body is what is known as the sixth sense, that is, the awareness of the inner sensations of the body: how the body feels internally. Therefore, when people are aware of their bodies they tend to be more compassionate and have empathy. Research shows that the more people are attuned to themselves, the more they are attuned to others and the more they are compassionate to themselves the more they are compassionate towards others (Siegel, 2010a). The seventh sense is the awareness of mental activities of thoughts, feelings, attitudes and intentions. All these are the sensations of the seventh sense which is mental activity. People can also observe themselves having the thought, they can also think about the concept of the thought they are having, conceiving the concept of the thought. A person can also have the knowing. Knowing can help them make a decision. The individual, however, must possess the ability to differentiate between sensing, observing and thought. These give filters through which rim elements can come into the hub of the WOA (Siegel, 2010a). The eighth sense is known as interconnectedness awareness. This awareness enables people to see each other as ‘we’. This is what is known as a ‘we map’ which allows people to accept and be aware that they are connected to others. It is a shared interconnectedness. Being aware aids response flexibility. People can move from probabilities to the openness of responses, and then to possibilities (Siegel, 2012). A compassionate communication between people appreciates their differences and promote their linkages. This fosters an integrated relationship with one other (Siegel, 2010b). Therefore, the WOA offers a holistic approach to conceptualising and practicing communication where awareness of both internal and external stimuli are brought into conscious awareness. In this research, Mindsight was introduced through the application of the WOA to the activity of email communication, resulting in the development and implementation of the Mindsight Utility for Virtual Communication (MUVC) via email (Appendix 1).

## 4 Research process

The planning of the research and the development and refinement of the MUVC via email, took eighteen months to complete. The research recruited two sets of participants: - a student cohort; - and a group of staff members of an International social entrepreneurs’ organisation. Ethical approval for this research was obtained from Northumbria University within the first year of commencement (a unique ethical number was given to the researcher by the University). Each participant was fully informed about the research project and were also told that they could withdraw from participation at any time. At all times confidentiality was maintained; unique identity numbers were given to the data of each participant. All required Northumbria University research ethics consent forms were signed accordingly. Original data entries were stored on the secure University drive for heightened security measures.

The research adopted an action research approach embedded within a case study in order to capture participants’ experiences (Denzin and Lincoln, 2011). It was considered important to gain access to the personal observations of individuals, to explore their experience of email communication.

The cycle for the action research is displayed in Figure 1 and the MUVC exercise can be found in Appendix 1.

The participants were asked to engage with the MUVC (Appendix 1) while conducting email for a period of six consecutive days. This required self-observation and reflection concluding into personal guidelines developed by each participant. Thus, the action research allowed self-ethnography to provide the means for interpretations of experiences by the participants themselves. This was recorded as part of the MUVC protocol. This process resonates with the ethos of action research giving the participants the opportunity to research themselves in social settings (Kemmis et al., 2014). The completed diaries (which captured the recorded experiences and personal guidelines) were analysed using thematic analysis.

Thematic analysis is a method used to identify, analyse and interpret the pattern of meanings in qualitative data. This provides an instrument that is not tied by paradigms but gives room for its adaptation. Thematic analysis emphasises the lead role of the researcher in formulating themes from the data that was obtained (Clarke and Braun, 2017).

The data from the diaries were analysed according to the predetermined categories of the WOA embedded in the MUVC protocol (Appendix 1). These predetermined categories are displayed in Table 1, the categories were further broken down into emerging themes (see second column in Table 1).

**Table 1:** Categories and main themes in the analysis.

Categories	Main themes
Sensory Awareness	Sight, hearing, taste, touch and smelling.
Bodily Awareness	Comfort and discomfort.
Mental activity Awareness	Emotions, intentions, thoughts and attitude.
Interconnectedness Awareness	Empathy, compassion and communication.

Many rounds of reading the completed diaries were performed to capture the emerging themes. First, a diary was read several times to understand and capture clues and meanings, then the emerging themes and statements where underlined. The underlined sentences and themes where categorised according to what, how and why things happened. These were captured and coded under the categories and sub themes using the NVIVO software.

## 5 Analysis

**Figure 2:**
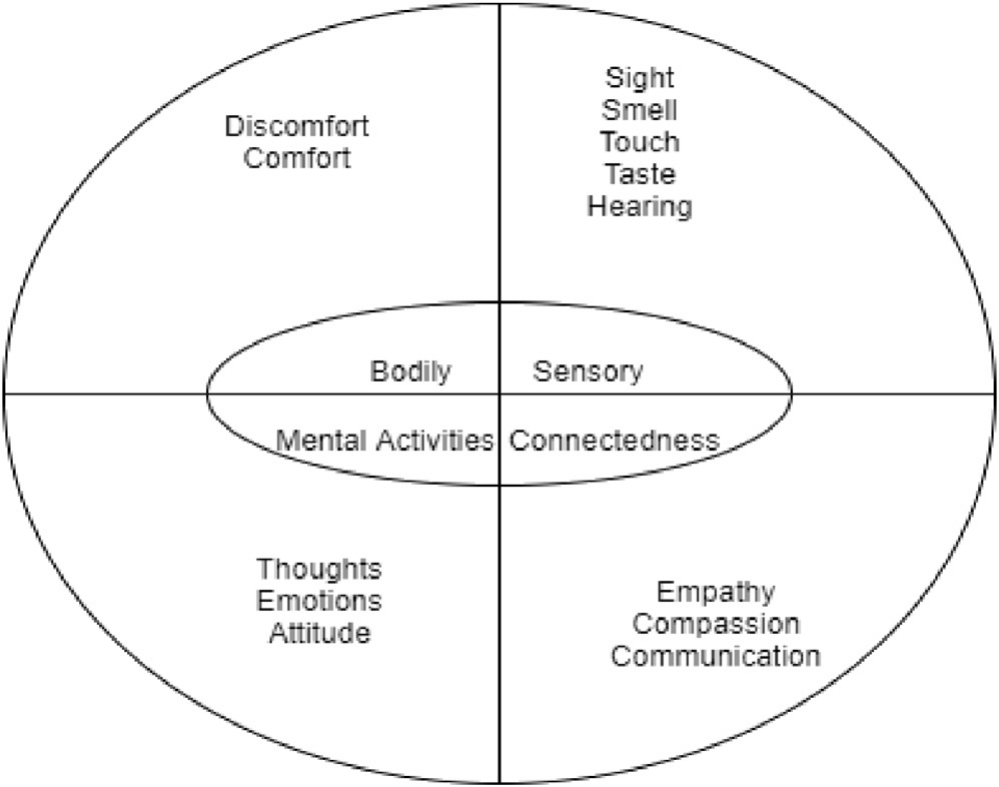
WOA used in thematic analysis.

The analysis began with a visual interpretation of data using the WOA circle. A WOA circle was drawn on a broad sheet of paper (A2), the circle had three layers. Then the circle was divided into four equal parts (quadrants) to represent the four senses of awareness, which are awareness of senses, the awareness of the body, the awareness of mental activities and the awareness of interconnectedness. Figure 2 shows the circle of the WOA for the thematic analysis. The first (inner) layer of the circle had predefined categories (themes) of sensory awareness, bodily awareness, mental activity awareness, and interconnected awareness. This was selected in order to investigate peoples’ ability to develop these categories of awareness.

The individual diaries were read to identify subthemes inside the main four categories (themes) When an individual's diary is read, the sentences that fall under a particular theme/subtheme are highlighted, then allocated to a theme/subtheme node (second layer of the circle in Figure 2).

A full representation of an individual thematic analysis is given in Appendix 2.

This paper presents the results from 19 diaries: six from the international social entrepreneurs’ organisation based in New York, USA and 13 diaries from a cohort of final year students in information technology management. The diaries of the participants were read and categorised according to the specific mental awareness that came into acknowledgement while doing email observing the MUVC practice. Starting from the first quadrant of awareness of senses, some participants seemed to be aware of their senses and were able to notice little things like the feeling of the computer keyboard beneath their fingers, which they mostly used to type out email communication. Some of the participants were touch aware which was felt from their skins (warm, cold and computer). Also, the intensity of the participants' alertness was displayed by the extent of their noticing the taste in their mouths. In addition, some participants notice the colour of the sky, and the background noise of the air conditioning system all at the same time.

*"I see the big grey sky through my window. I see a small lamp with yellow light. I smell food from my breakfast. I'm touching the keyboard of my laptop. I taste my usual mouth taste. I hear the ventilation system in the building, the cars in the street." Participant LJ.*

*"I see the aftermath of a party, the smell of delicious tacos, got to hit the piñata at the holiday party, can still taste the tacos I ate before." Participant FM.*

**Figure 3:**
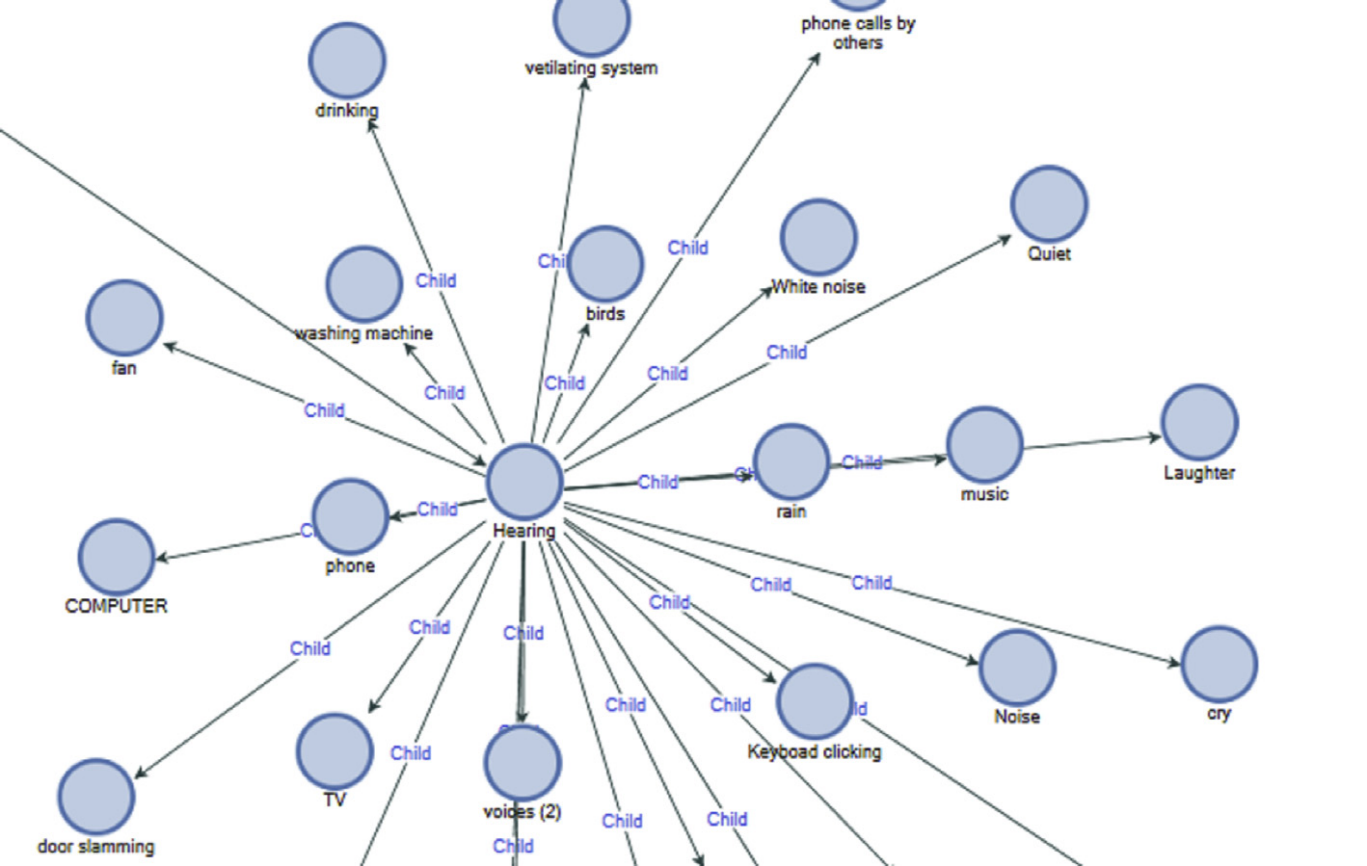
Sound awareness.

Figure 3 shows the various sounds people were aware of.

**Figure 4:**
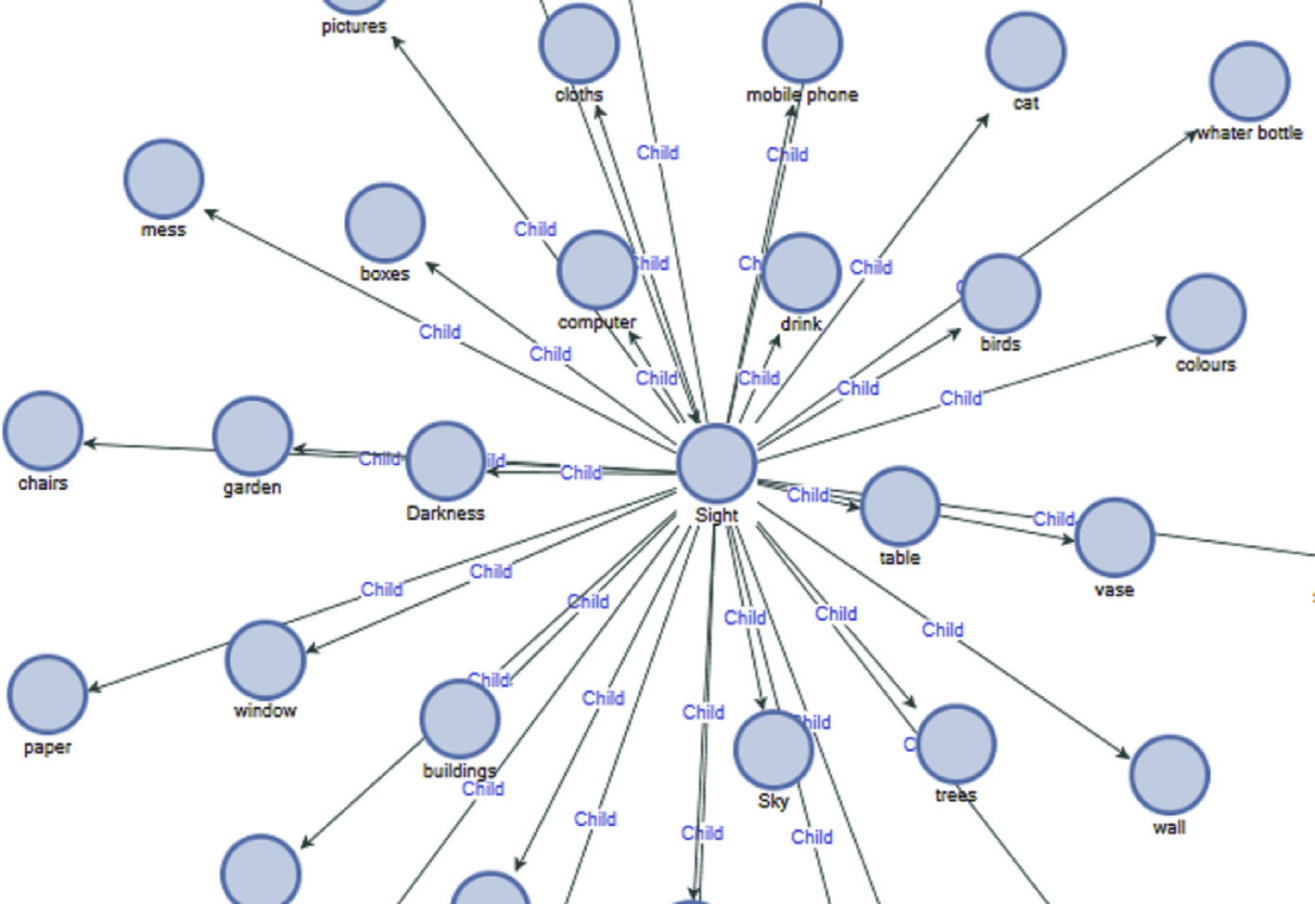
Sight awareness.

Participants were aware of other sounds like birds, cat, clock, conversation, drinking, laughter, music, noise, phone, plane, quiet, rain, traffic, ventilating systems, and voices. Also, other items that came into sight awareness were, birds, boxes, buildings, cats, chairs, colours, computers, dishes, dogs, drinks, flowers, fruits, laundry, light, mobile phones, mug, notebooks, people, screen, sun, table and vase water (See Figure 4) This demonstrates that an individual can be awakened to all sensory inputs and they have impact on experience.

### 5.1 Bodily awareness

It was also revealed that, people could form bad postures when they are overwhelmed by numerous task that demands their attention. The same level of alertness draws attention to the feeling of pain. For instance, clothes that are uncomfortable and the feeling of cold could cause distraction during email communication. Additionally, being nervous comes with some physical symptoms even though it starts from the mind. The symptoms include tensed shoulder, hands and forehead. Not only does being nervous and anxious cause problems emotionally but also physiologically. However, some participants found a way of dealing with email stress by taking a break. This was achieved by allowing some time to pass before replying to emails without the pressure and at another time taking a holiday altogether. Sometimes the sight of a landscape can induce a relaxing feeling. Also, working in a familiar environment with the sun shining through gives a feeling of relaxation.

### 5.2 Mental activity awareness

People experienced emotions while engaging with email communication for instance, people felt annoyed when they are always asked to do the task that belongs to others. This breeds frustration that results in anger. Other times, it is questioned in the form of an email that suggests to the individual that they are expected to do a task which is not their responsibility. Some of the frustration is fueled when there are no clear-cut duties among staff members so that the work subtly returns to the person who is always happy to help. This reduces the individual's productivity and as a result of being overloaded by other peoples' tasks.

*"Intention on emails is to solve the problem, but my feeling is that instead of trying some people just ask me to solve, without providing the tools for me to help, that frustrates me even when it comes from a good intention, I understand that solving many problems look good but are they actually solving them? Or just sending emails for others to solve? Responsibilities are not clear and that tires me." Participant FM.*

**Figure 5:**
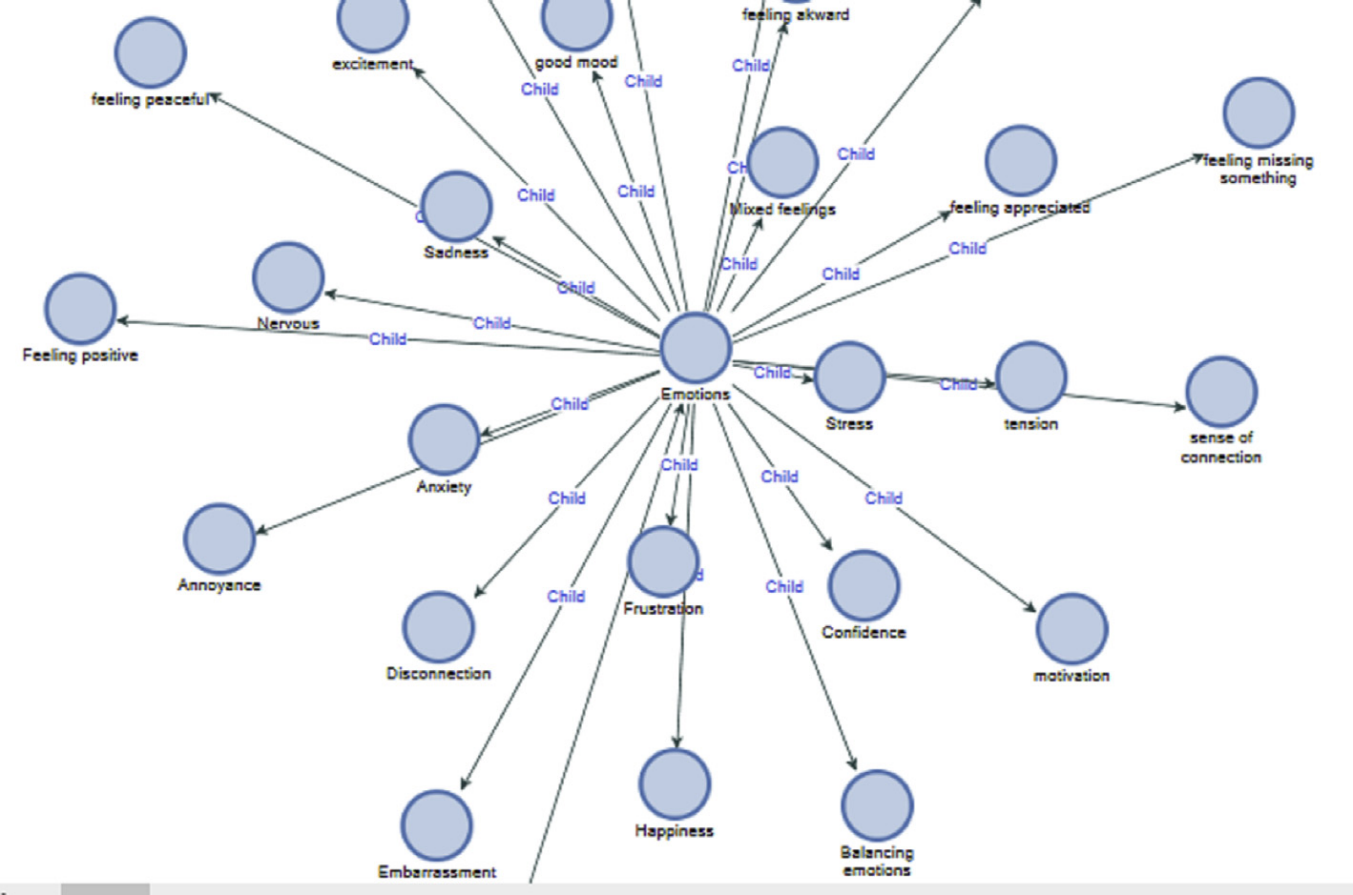
Emotions awareness.

Again the power of emails on emotions is presented, when another individual felt happy after receiving a pleasant email from someone. This shows that the content of an email affects the emotions and vice versa (Figure 5).

It is possible that an email could both make a person to be happy and angry at the same time; however, the anger can be minimised if the sender uses a professional and calming tone.

Sometimes, negative emotions present as anxiety, stress and tension develop when emails are received from superiors with whom people have complicated relationships. This shows that email could have a stronger influence than presence. The participant might not have the sender in the room, but the impact of his/her email is already felt in the participant's body so that it caused some tension and stress on the participant. Other times anxiety beclouds focus and causes a distraction. This emphasises the impact email communication has on people's physiology and psychology. Having spam emails or generic emails tend to give people the feeling of being overloaded:

*"The last email that I have received today is just an automated email stating content has been uploaded to blackboard for a certain module. Whenever I get these automated emails, I do feel a certain amount of pressure to do the work related to the update as fast as possible. These emails create a negative response because they make me feel like I am not able to keep up with all the information and workload of the course.” Participant TMFB.*

Further findings from the participant suggests that email has both physiological and psychological impact on its users. For instance, some participants felt connected because colleagues tried to help or assist with work, this kind of commitment from others also gave a sense of being appreciated. While others felt happy when good news came from an email and anxious as a result of other emails that had compliant.

*"A second email made me feel excited as one colleague had joined one of my projects. At that moment, other colleagues came into the office, and I got distracted. Then, I got an update from a team member commenting on a couple of emails that I am expected to read and that made me feel anxious given that the two messages were complaining. I'm exhaling longer now." Participant FM.*

People can manage their emotions when they can notice their emotions and can take the next cause of action. Moreover, that is what Mindsight is about, that people are not carried away by their emotions but can directly channel them appropriately. Therefore, the participant noticed his/her feelings and took control of it.

*"I'm feeling a bit rushed and with no time to do this exercise. I just want to go ahead and work. Trying to use my breath to let go of that feeling/thought." Participant LJ.*

Additionally, too many emails tend to create thoughts of overload. This might cause stress and pressure; this also translates into an individual's physiology as participants reported being slouched.

*"Too many emails and Skype calls and messages, need some time without solving other's problems to start working on my projects, thought this week was going to be easier but there's a lot of work, I slouch." Participant FM.*

**Figure 6:**
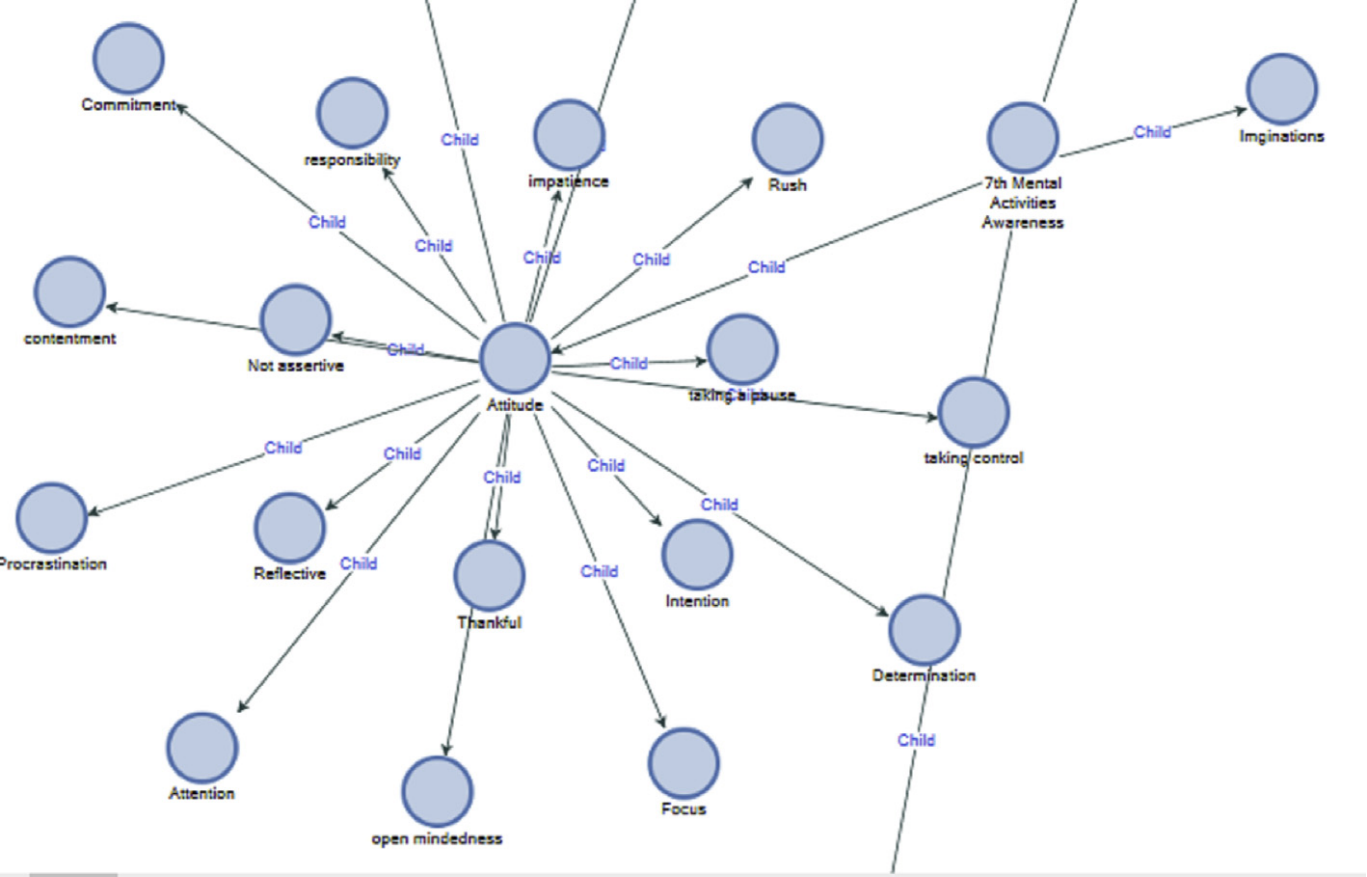
Attitude awareness.

Other types of mental activity awareness manifested in peoples' attitudes. This revealed how people react or decide to deal with email communication in different situations (Figure 6).

### 5.3 Interconnectedness awareness

Interconnectedness awareness is in the fourth quadrant of the WOA which makes up one of the categories for the thematic analysis. This category is further broken down into three other main themes. They are communication, empathy, and compassion.

**Figure 7:**
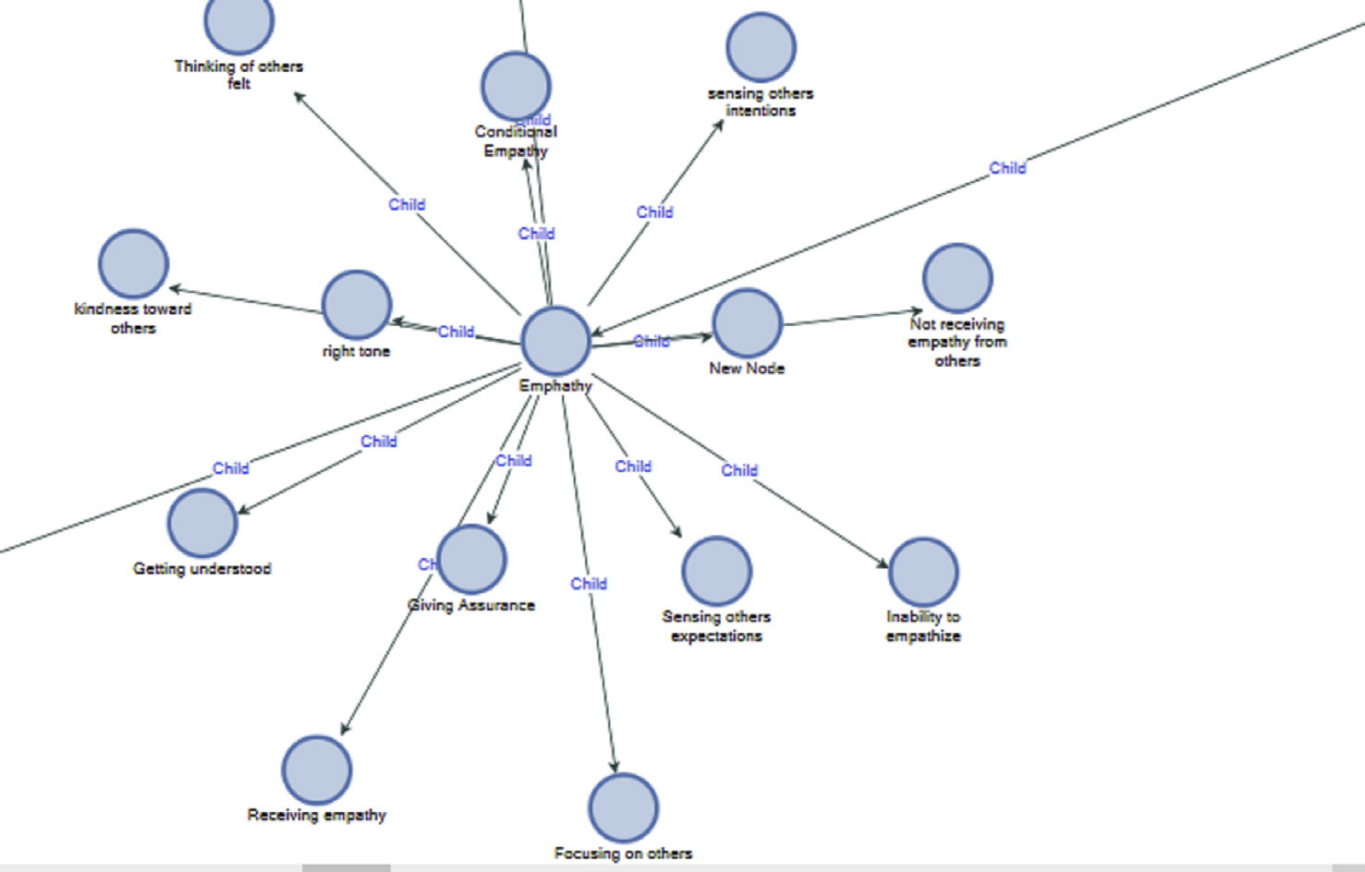
Empathy awareness.

Interconnectedness is the awareness people have about other people and how a connection with other people is achieved through communication, empathy, and compassion. Sometimes empathy is shown by reassuring another person (see Figure 7). For instance when an individual tries to understand the situation of others as to why they behave in a certain way, then the person is giving empathy. While giving the empathy, they are careful with the way they communicate so that they do not project negativity unto their receivers.

*"Their replies appear to be quite rigid, perhaps they are under a lot of stress and pressure themselves. They may well be working within a very tight procedure/ process when trying to resolve such issues as the ones that I have raised...I am trying to resolve the issues that I have had via email. I am doing so by making sure that the way that I am coming across in email is not seen as aggressive or too negative, despite my frustration.” NNFB.*

When mistakes occur, it is empathy and understanding that helps individuals to respond appropriately to the situation without being judgmental of others. Giving empathy to someone that stops you from giving negative responses. As the saying goes, you can quickly change your mood, but you will not be able to change what you said.

*"The sender of an email telling me information on one module appears to be rushed, and probably stressed. There are repeated requests in the email to avoid nagging certain lecturers in regard to grades. There are spelling mistakes in the email, leading me to think they're rushed. I can relate to the feeling of needing to send out emails (and complete tasks in general) as quickly as possible, and therefore occasionally making mistakes (such as spelling mistakes). I am hoping that the follow-up email with grades is sent to me soon to stop anticipation. I have recently been sending messages out to members of a group very quickly, so understand the pressure to inform people accordingly." NN2FB.*

Showing empathy to someone might involve thinking about the choice of words not to complain to them about some shortcomings, but constructing words that will convey an understanding of the situation they are in. such messages are well received, because, when a receiver feels that the message sender has empathy for them, they feel connected to them.

Other times participants reported being open-minded toward working with colleagues. For example, an individual was happy to go along with a colleague because of a shared goal. It reveals that the person was ready to link differentiated parts in the sense that, they have different ways of thinking but want to work together in order to achieve an important goal. This shows that communication moves in a positive direction when people are friendly and willing. People have an agreement when they value the relationship, they are in with the person they are communicating with, and so they are happy to make sacrifices.

*"One colleague, who I've been expecting to hear from, has sent me a message. Her response can go one of two ways, and I am nervous to open her email. I'm in defense-mode, we need to finish something together, and she's only sent me an email on Sunday afternoon -this is late, if we won't make it, it's her fault. When I open the email: prospects are good, we'll get this done today! I worried for nothing, which causes a great sense of relief. This affects my mood significantly. Most likely it influenced my decision to partake in this exercise. This also seeps through in my response to her and to the other people I am writing to today. There's a cheerful undertone; I am friendlier and more willing to do them any favours than I otherwise might have been" Participant OB.*

Although misunderstanding can occur when the receiver does not understand what the email requires, therefore, there will be a sense of getting lost in the loop, and as a result, the action required by the sender might not have been achieved. (it is crucial for senders of email to write in an understandable language so that the receivers can understand what has been required of them).

*"My first email. I don't understand what I'm asked to do. Feel a bit lost. It has some links I clicked and that got me into other random links pages. It distracted me and took me to my personal email"…Got distracted again and looked at my cellphone." Participant LJ.*

**Figure 8:**
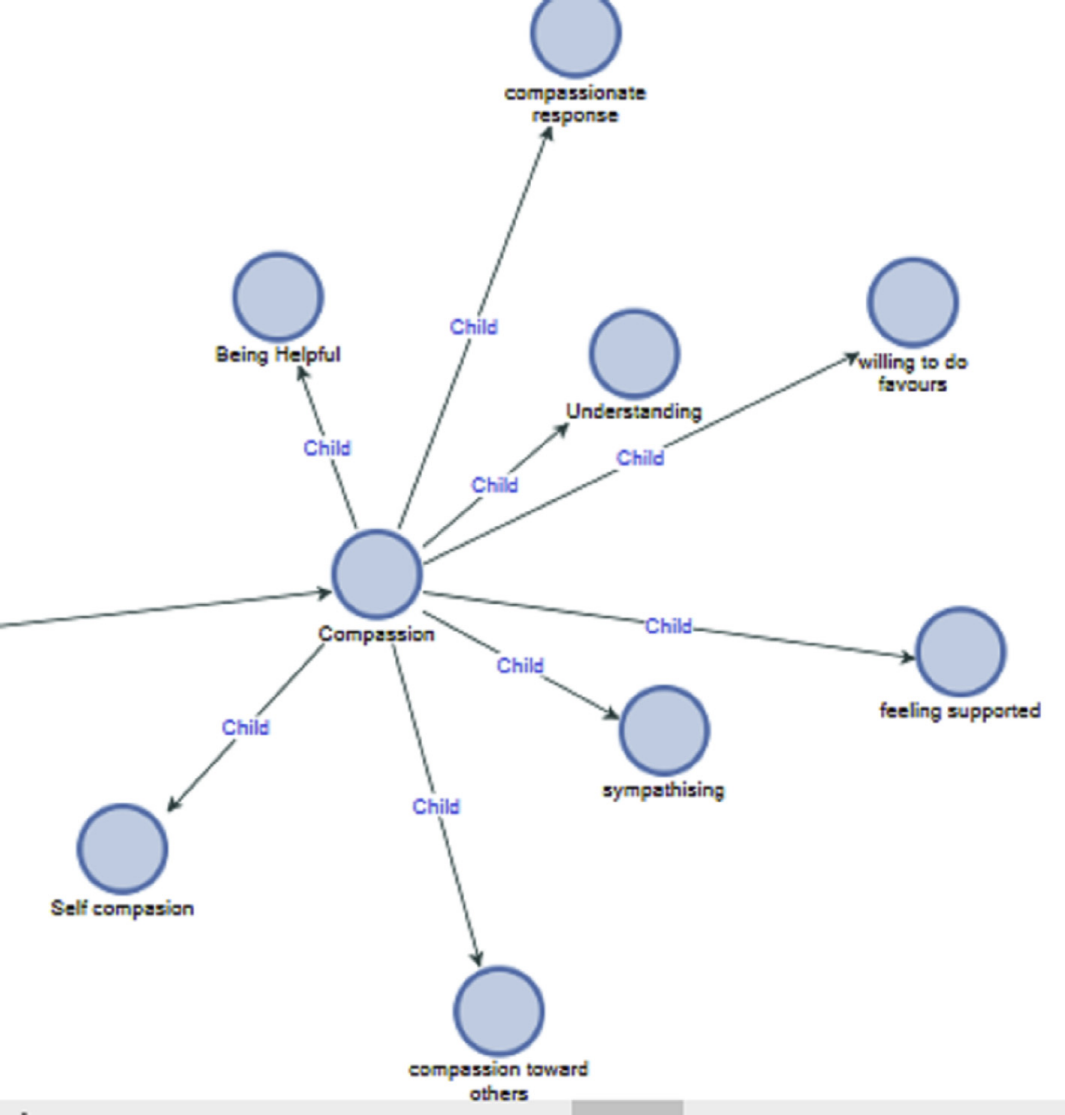
Awareness of compassion.

This might imply that there is a connection between not understanding communication and been distracted. This made the participant further develop an attitude of disengagement. The solution could be considering context. Compassion related experiences were understanding, sympathy, self-compassion and compassionate response (see Figure 8).

## 6 Discussion

The findings of this research suggest that the application of Mindsight has the potential of addressing some of the issues of email communication. The analysis of the participants experiences suggest that what comes into people's awareness could either encourage or hinder their abilities to communicate effectively. Categories of facilitators and inhibitors of email communication are presented in Table 2.

**Table 2:** Facilitators/inhibitors of virtual communication via email.

Awareness	Facilitators	Inhibitors
Sensory Awareness	• Music	• Cry
	• Quiet	• Noise
	• Light	
Bodily Awareness	• Breathing	• Back discomfort
	• Feeling comfortable	• Stress
	• Good posture	• Heat
	• Seeking comfort	• Hunger
	• Stress relief	• Illness
	• Taking breaks	• Negative energy
Mental Activity Awareness	• Open mindedness	• Procrastination
	• Attention	• Not assertive
	• Focus	• Annoyance
	• Gratitude	• Anxiety
	• Positive Intent	• Disconnection
	• Reflective	• Embarrassment
	• Determination	• Frustration
	• Calmness	• Sadness
	• Confidence	• Wondering mind
	• Feeling positive	• Distraction
Communication Awareness	• Empathy	• Conditional empathy
	• Focusing on others feelings	• Inability to empathise
	• Getting understood	• Communicating impatience
	• Positive response	
	• Right tone	
	• Kindness toward others	
	• Clear language	
	• Compassion toward others	

Facilitators of email communication are the conditions in people's awareness that encourages email communication. Inhibitors are hindrances to the communication process. It is important to recognise that these categories are context and participant dependent and cannot be generalised as individuals will have different preferences and sensitivities and these will also vary with different circumstances and contexts.

The results show that different groups of participants exhibit differences, i.e.: The students reported distraction by people, animals, noise and mobile devices more often than the professionals; -The professionals were often concerned how their action will impact others; -The intent to understand and seek collaboration was pronounced stronger in the professionals group.

**Figure 9:**
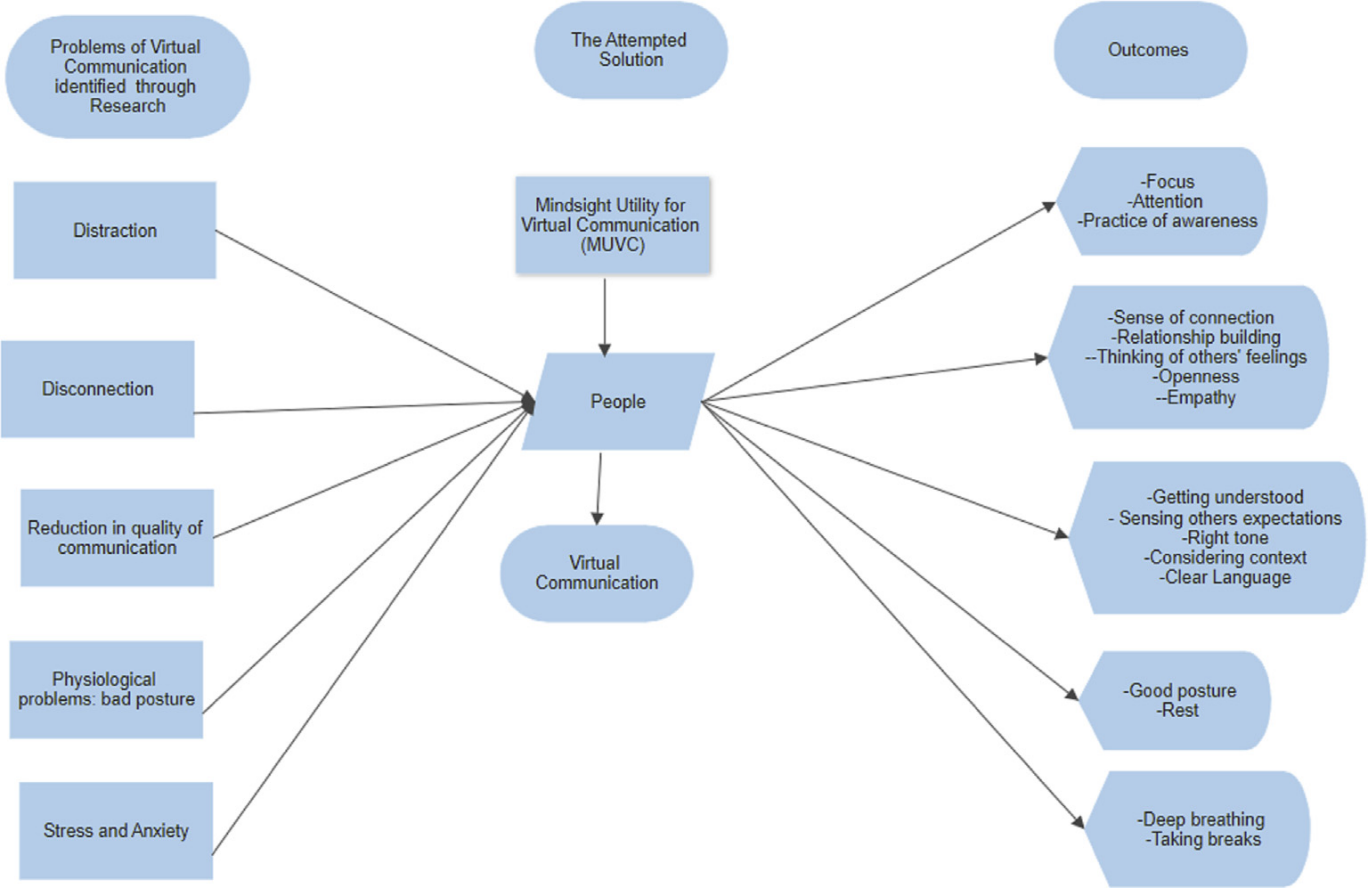
Solution strategies to Communication Problems in Email.

The practice of the MUVC showed some possible strategies of addressing email communication issues. Figure 9 depicts how the application of Mindsight within email could potentially address these issues. The first column represents the problems (issues) identified by the participants in this study, while the second column depicts the Mindsight process. The third column displays the extracted solutions and strategies reported by the participants. Examples of these strategies in addressing the issues, are given in the following sections.

Having an open-mind and empathy tackled the difficulties in connecting with others virtually due to differences in background. From the evidence gathered it was discovered that, a possible way to connect with another person from a different background from you is to empathise with them, i.e. trying to view the world through their own lens.

For instance, when an individual tries to understand the situation of others as to why they behave in a certain way, then they are giving empathy. They are careful with the way they reply messages and seek to understand others.

*"Their replies appear to be quite rigid, perhaps they are under a lot of stress and pressure themselves. They may well be working within a very tight procedure/ process when trying to resolve such issues as the ones that I have raised...I am trying to resolve the issues that I have had via email. I am doing so by making sure that the way that I am coming across in email is not seen as aggressive or too negative, despite my frustration.” NNFB.*

Lack of trust and disagreement was addressed by building relationships with the people virtually and also developing a sense of connection with them. When people are helpful, it is demonstrated through solving problems for others which leads to trust. Then trust leads to the feeling of interconnectedness which establishes relationships.

*"I prioritize problem-solving because of that feeling, coworkers know they can count on me for support and help, that level of connection and trust is very important for me." Participant FM.*

*"I want to help, offer everyone in the office may help, want to give, want to spend more time with them, want to share more and make them feel valued from the Global office." Participant FM.*

Another aspect of addressing disagreement is that the person might choose to think about the choice of words by constructing words that will convey an understanding of the situation they are in. This process reduces disagreements and misunderstanding.

*"I send an email to the head of department regarding the CIS building access issues. I wonder if I should contact someone else further down the hierarchy first about this - maybe the university's Ask4help service. However, I justify my decision to email the head of the department as he encouraged students to contact him with any questions about the new building. I sense that he is probably busy and stressed about the new building since it has been delayed so much and caused a ripple of issues such as timetable changes and other problems I won't even know about. I word the email to inform him of the issue, not to demand a quick fix. I don't express any of my frustration in my email; it is not his fault, so that would be unfair. I appreciate how much has been done for the students to make up for the delay, so I can understand that there will be some teething issues. I feel less frustrated thinking about it this way." FSFB.*

When a receiver feels that the message sender has empathy for them, they immediately feel connected to them. A sense of connection builds trust.

*“I have received an email from my dissertation tutor Jackie; she has sent a friendly email reminding me of the tasks that I should have competed for my dissertation. Although her email is friendly and light hearted, it is causing me a greater sensation of anxiety in my stomach because of the dissertation work that I need to complete. I feel a connection with Jackie as her email is very supportive and it is clear that she wants me to achieve a good mark with my work.” JGFB.*

Sometimes empathy is shown by reassuring another person. For instance, the participant gave another person assurance while acknowledging the person's concerns.

*“I am communicating with a friend who is also helping to organise the fundraising ball. I sense that my friend is also excited about the upcoming ball but at the same time is nervous since it is her first time organising a large event. I reacted warmly to my friend and reassured her that the event would run smoothly and that a lot of the organising is already done. I also reassured her that many people are helping in the organisation that can support her. My friend's reaction has furthered my excitement for the ball, but I am feeling more anxious after discussing her concerns as I feel that I have taken ownership and shared responsibility for her concerns. However, I feel better within my inner state for reassuring and supporting a friend.” Participant ABFB.*

It was also discovered that some participants have achieved deeper levels of integration (for an individual to achieve integration the person must have an open mind to receive other people's views and characteristics, by possessing the flexibility to accommodate people with their views and also be willing to work wholeheartedly together).

*"Just read one of the emails. Comes from a man I appreciate. We have different points of view about something we both care deeply. Working with him is a bit challenging because from my perspective he uses his intellect and I use my emotions more to guide me. I'm learning how to collaborate with people who have different a different way to do things but share a vision with me. I don't know if I can collaborate with someone who does not share a vision with me. My emotions are great here. I felt triggered, I had to breathe and let go. I had to wait and contain a bit. I still chose to share what felt important to me. Kept breathing". Participant LJ*

The lack of interpersonal clues was addressed by giving assurance, thinking of how others will feel, sympathising and getting understood; because in the absence of interpersonal clues one can only communicate in a clear language and select words that would convey what the person is feeling or expecting. Therefore, when you can read between the lines, you might be able to sense and understand the intentions of others and that in a way promotes relations and connections.

*“I sense that the person's intentions are kind and polite, I notice this due to the style of writing used within the email. The nature of the email makes me want to reply to the email in order to reciprocate this back to them in order to build a connection with them”. Participant KAFB*

*I have had some replies from ones that I have previously followed up on and they have been quite abrupt replies, which I understand, and also feel guilty as I am only adding pressure onto the individuals that have received my emails. I feel that the people that have previously replied intend to try and keep time spent on emailing students in regards to grades, such as me, to a minimum.” NN2FB.*

Deficient communication quality was addressed by ensuring that the context of communication was understood, using the right professional tone. This kind of disposition ensured that the communication was well received with the clarity required.

Physiological problems were handled by ensuring comfortable upright posture while communicating virtually. Relieving stress is addressed by taking breaks and balancing emotions. That way, both physical and internal wellbeing is taken care of.

*"I'm email thinking about new projects I have in mind and trying to make time to start executing them, too many emails and skype calls and messages, need some time without solving other's problems to start working on my projects, thought this week was going to be easier but there's a lot of work, I slouch." Participant FM.*

Bad postures induced by email stress were demonstrated throughout the diaries of individuals. There is a possibility of an email received to have such an effect on a person's physiology. It automatically pushes them to the point that they unconsciously slide their bodies into an uncomfortable position.

*"I got an email from a senior leader who triggers me very easily. Got a bit worked up before opening it. I took a breath and open. It was a neutral email. Just action done. I was nice in my reply. But feel how my body gets tense and my breathing gets shorten." Participant LJ*

Relaxation was combined with regulating breathing

*“I feel tired but relaxed this morning, there is no weight on my shoulders, which is making me calm. My breathing is still in sync, and I am ready to open my emails”. Participant JGFB.*

The body felt relaxed because of good posture.

*“Currently feeling very relaxed and content. Yes because of my straight posture and shoulders feeling relaxed I recognized once I had finished observing my surroundings I released my breathing.” Participant RBFB.*

Discomfort may be recognised when people get a sense of awareness of their lousy posture when their bodies begin to ache, and that awareness prompts them to adjust their posture.

*"I am not able to hold my breath as well as I would like due to me attending to problems via email, such as outstanding tasks that I need to resolve (such as refunds/ customer support issues that I have raised). I am aware that I currently have a bad posture, hence my back hurting." Participant NNFB.*

It is premature, on the basis of a limited study, to try drawing definite conclusions about the impact of the application of Mindsight on email communication and the effectiveness of the Mindsight Utility in developing self-awareness. Thus, all generalisations apply only to the group of participants in this research. There is a need for further studies to explore the usefulness of the approach with a larger number of participants and in variety of contexts. It is also important to consider appropriate metrics of the impact of the practice on the email communication, from both first and third person perspectives.

## 7 Conclusion

The theory of Mindsight was examined with regard to developing practices of self-awareness and refection in email communication. MUVC via email was developed and implemented. An exploratory study with two groups of participants was conducted to examine the impact. The study in the application of MUVC to email revealed potential problems and solution strategies, inhibitors and facilitators of communication. Problems with email were perceived as: not knowing one another, difficulties in connecting, lack of trust, lack of interpersonal clues, reduction in communication quality, emotional and psychological discomfort. Solution strategies included: open-mindedness, empathy, compassion, attention focus, clear language, awareness practice, addressing physical and emotional discomfort.

Further studies are needed to evaluate the significance of the MUVC on developing self-awareness, awareness of others and effective strategies in email communication. There is a challenge, i.e. how do we motivate participants to engage in the practice in larger numbers? A potential answer would be to seek insight from behaviour theories. The Theory of Planned Behaviour (Ajzen, 1991) offers practical insight on how personal attitude (rational and emotional), social encouragement and perceived control of behavior, impact the intention and adoption of the behaviour. Further research into: -what people think about the MUVC; how they perceive its use by others; how they perceive its value to themselves; -whether they can perform the behaviour; and how they can implement it, would inform ways of wider adoption on practice.

## Declarations

### Author contribution statement

S. Ogwu, P. Sice, S. Keogh: Conceived and designed the experiments; Performed the experiments; Analyzed and interpreted the data; Contributed reagents, materials, analysis tools or data; Wrote the paper.

C. Goodlet: Analyzed and interpreted the data; Wrote the paper.

### Funding statement

This research did not receive any specific grant from funding agencies in the public, commercial, or not-for-profit sectors.

### Competing interest statement

The authors declare no conflict of interest.

### Additional information

Data associated with this study has been deposited at Northumbria University Drive.

### Additional information

No additional information is available for this paper.

## Appendix A Supplementary data

REVIEWED MANUSCRIPT.docx
