# Supplementary material for: An exploratory study of the application of mindsight in email communication
Source: Heliyon. 2020 Jul 15;6(7):e04305. doi: 10.1016/j.heliyon.2020.e04305 (PMC7364031; doi:10.1016/j.heliyon.2020.e04305)

## **Appendix 1 Email Observation Practice used with the participants in the research**

## **Mindsight Utility for Virtual Communication (MUVC) via email:**

Do your normal email activity and do this “primary practice” once a day for twenty minutes for several days, or preferably for an entire week. For the period devoted to this practice, you can limit yourself just to email, artificial though this may feel; or, you can maintain your normal multitasking behavior, but focus your attention primarily on your email. The emails you should focus on, need to be the ones, you received from people directly, not generic emails from your organization or institution.

*The Intention is to Observe What you are Doing and Feeling.*

Please log the time and place before you begin: Are you at home, bus, sitting in a noisy coffee house? What time is it? Keep a diary and record your observations during this practice.

**Now follow the steps:**

**Step 1. Begin by getting into a comfortable position** with your feet firmly on the floor, your spine straight with shoulders relaxed, you may choose to move and adjust posture until you feel comfortable. Do not slouch. Take a couple of deep breaths, exhaling slowly through your mouth.

**Step 2. Now, turn your attention toyour immediate environment**. What are you seeing, hearing, smelling, touching? Any taste in your mouth? ***Log what you are observing in your diary.***

**Step 3. Next, focus your attention on the inner sensations of your body** Do you now notice whether you feel relaxed and content, or tense and anxious? Are you suddenly aware that you’ve been holding your breath? Any sensations in your body?  ***Log what you are observing in your diary.***

**Step 4. Now, turn your attention to any mental activity**: intentions, images, attitudes, thoughts, feelings. First, begin by just becoming aware of what enters your mind Spend a few moments getting to know what arises in your mind. Notice, how it comes about, and when it subsides. Are you aware of your state of observing what arises? Keep your spine straight and shoulders relaxed. ***Log what you are observing in your diary.***

**Step 5. Now focus your awareness on the people who you are communicating with** (receiving or sending email).

Spend a few moments to sense their intention, mood, expectations, desires, etc. Be patient with yourself, focus on developing an intent to connect with the person you are communicating with. Observe your own reactions, sensations, feelings, thoughts, desires. Any sense of connecting/disconnecting with the other person? How is this related to what you’ve just been doing or thinking? Keep your spine straight and shoulders relaxed. ***Log what you are observing in your diary.***

***Step 6: Consolidate (summarize) your Observations***

Now read through your notes and reflect on them. You might think that this step is unnecessary, but it is actually quite important. Looking back over everything you’ve written will give you the chance to notice patterns that may not have been obvious in the moment, and to fill out your understanding by comparing and contrasting multiple observations. Are there times when you use email as a distraction from other more important activities? Why do you check email when you do, and how well does this work for you? Does it make a difference whether you are checking email on your computer or on your phone?

This is where paying attention to your immediate experience can really pay off. When your email practice is problematic (whatever that means for you), you may find that you have been holding your breath, or collapsing your chest, or feeling anxious or upset. And when your email practice is going well, you may find indications of that in your mind and body-a greater sense of relaxation, a lightness of mood, easiness of communication, etc.

***Step 7: Formulate Personal Guidelines***

The summary remarks that you’ve just created will give you good sense of what is currently working well and what isn’t. This understanding provides the basis for making useful changes, which you should now formulate as personal guidelines for future behaviour. These guidelines are personal: they are particular to your observations and habits. They don’t need to work for anyone else. What’s more, they are provisional: subject to further change as you discover more about yourself, or as your circumstances change.

Broadly speaking, there are two kinds of changes you might consider making to your online practices (or what you do) and to yourself (to how you are). When you see that there are changes that can be made, that will improve your effectiveness and connectedness with others- for example, by reflecting on your responses, emphasizing with others, or by limiting the number of times you check email during the day- then by all means make them. In this instance, you might write a guideline that says, “deal with email no more than three times a day”, “pay attention to emotions and how this may affect communication.

But if you find that you can’t change the external conditions, because, for example, your job requires you to be online and continuously handling a torrent of email messages-you still have the possibility of changing the way you deal with these circumstances *inside yourself.*

Thus noticing the resistance, you feel to handling all this email and how it affects your body and your emotions, you might decide to reduce your stress by acknowledging what you’re feeling and making a conscious effort to relax in the face of torrent.

**Appendix 2. Thematic analysis example**


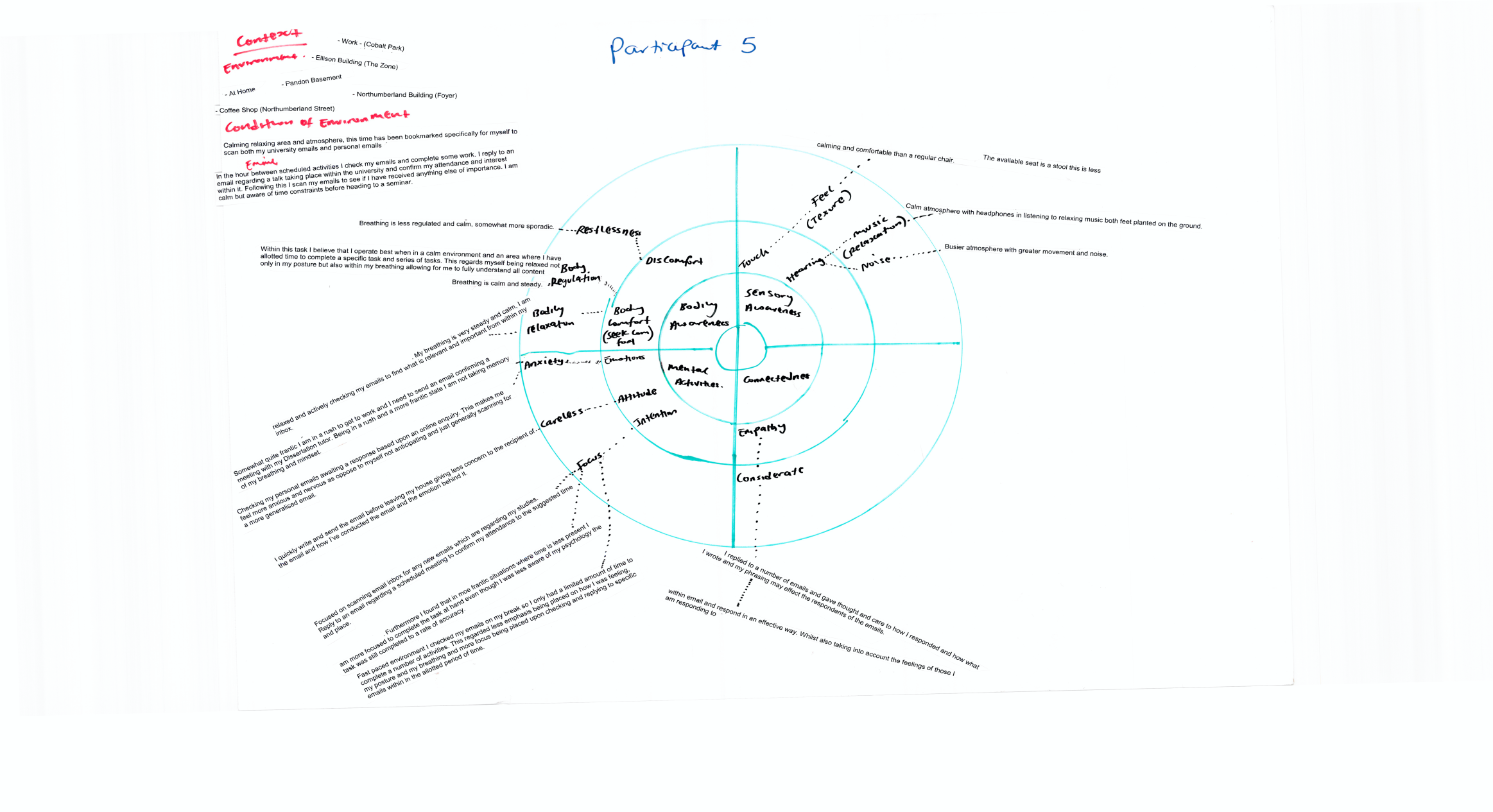

Supplement: Supplementary file 1 — REVIEWED MANUSCRIPT.docx [file mmc1.docx]
